# Supplementation with Proline Improves Haemato-Biochemical and Reproductive Indicators in Male Rabbits Affected by Environmental Heat-Stress

**DOI:** 10.3390/ani11020373

**Published:** 2021-02-02

**Authors:** Sameh A. Abdelnour, Naif A. Al-Gabri, Nesrein M. Hashem, Antonio Gonzalez-Bulnes

**Affiliations:** 1 Department of Animal Production, Faculty of Agriculture, Zagazig University, Zagazig 44511, Egypt; samehtimor86@gmail.com; 2 Department of Pathology, Faculty of Veterinary Medicine, Thamar University, Dhamar 2153, Yemen; naifaljabry@yahoo.com; 3 Laboratory of Regional Djibouti Livestock Quarantine, Abu Yasar International Est. 1999, Djibouti; 4 Department of Animal and Fish Production, Faculty of Agriculture (El-Shatby), Alexandria University, Alexandria 21545, Egypt; 5 Departamento de Reproducción Animal, INIA, Avda, Puerta de Hierro s/n, 28040 Madrid, Spain; 6 Departamento de Produccion y Sanidad Animal, Facultad de Veterinaria, Universidad Cardenal Herrera-CEU, CEU Universities, Tirant lo Blanc, 7, Alfara del Patriarca, 46115 Valencia, Spain

**Keywords:** proline, antioxidants, heat stress, metabolites, semen, rabbits

## Abstract

**Simple Summary:**

The exposure of rabbits, such as other mammals, to environmental heat stress drastically affects homeostasis and reproductive function. The current trial indicates that a dietary supplementation with 50–100 mg/kg DM of proline improves redox status, blood metabolites, and reproductive traits of rabbit bucks.

**Abstract:**

Twenty-four adult rabbit bucks (*n* = 6 per treatment) were fed a basal diet supplemented with 0 (control), 50, 100, and 150 mg proline/kg dry matter (DM) diet for 12 weeks to determine possible usefulness for alleviating the negative impact of environmental heat stress on redox status, haemato-biochaemical attributes and semen quality. There were significant dose–response effects, with increments in levels of dietary proline (LDP) quadratically improving red blood cell counts (*p* = 0.017), rectal temperature (*p* = 0.009), and respiratory rate (*p* < 0.001). Increasing LDP cubically affected superoxide dismutase activity in blood plasma (*p* = 0.012) and total antioxidant capacity in both blood and seminal plasma (*p* < 0.001 and *p* = 0.006, respectively). The optimal response was observed at 30 and 80 mg proline/kg DM for blood and seminal plasma, respectively. With regards to homeostasis indexes, increments in LDP cubically modified blood plasma concentrations of total protein (*p* = 0.002) and albumin (*p* < 0.001), with an optimal response found at 70 mg proline/kg DM. A linear relationship (*p* = 0.005) was also observed between LDP and blood plasma glucose concentrations, with the optimal response being found at 100 mg proline/kg DM. Increasing LDP also showed positive effects on reproductive traits, with quadratic increases in blood plasma testosterone and cortisol concentrations (*p* < 0.001; optimal responses at 50 and 60 mg proline/kg DM, respectively), a positive linear relationship with in libido, ejaculate volume, sperm concentration and total sperm count (*p* < 0.001 for all; optimal responses observed at 100 mg proline/kg DM) and a quadratic increase in total functional sperm fraction (*p* < 0.001; optimal response at 70 mg proline mg/kg DM). Hence, the optimal positive effects of dietary proline supplementation on redox status, blood metabolites, and reproductive traits of rabbit bucks may be achieved at 50–100 mg/kg DM.

## 1. Introduction

The exposure of mammals such as rabbits to environmental heat stress drastically affects homeostasis. Heat stress alters animal behavior, causes metabolic/hormonal imbalances, and promotes free radical production, leading to inadequate productive and reproductive performances [1,2]. In rabbits, heat stress can stimulate drastic changes in behavior and biological functions, including depression in feed intake, disturbances in several metabolic processes (water, protein, energy, and mineral balances), hormonal secretions (e.g., metabolic and sexual hormones), and blood metabolites. In rabbits, exposure to temperature humidity index (THI) 30 or more completely disrupts all thermoregulatory mechanisms and evokes certain elevation in the hypothalamic physiologic set point [2]. Heat stress can also impair sexual behavior (libido) and semen quality traits in terms of motility, viability, and morphology parameters and metabolic activity [3]. As a result, multiple thermoregulatory defense mechanisms are stimulated at physiological and cellular levels to achieve thermostasis and counter the negative impacts of heat stress. Studies to identify cellular defense mechanisms against environmental stresses implicate the amino acid proline and its metabolites as components of cellular defense responses under stress [4,5]. 

Proline is an amino acid with a unique chemical structure and multiple biological functions; it acts as a proteinogenic amino acid, an osmolyte, and an antioxidant [4,6]. In addition, proline regulates several pathways that are active under various stress conditions [7]. Among these pathways, proline dehydrogenase/proline oxidase (PRODH/POX) signaling is upregulated under oxidative stress and other stress conditions [5]. Proline also regulates the mammalian target of rapamycin activation pathway [8], which integrates signals regarding nutrients (glucose and amino acids), cellular energy status, and growth factors, thereby controlling cell proliferation and function [9,10]. Kang and coworkers [11] found that dietary supplementation with 1% proline increased the expression of heat shock protein 70 (HSP70) in weaned piglets that were challenged with lipopolysaccharide. In adults, Ma et al. [12] observed a relationship between decreased blood plasma proline concentrations and increased prevalence of semen abnormalities and male infertility. Accordingly, proline can be considered a functional amino acid for mammalian species [13]. In most mammals, proline is classified as a nonessential amino acid, although under conditions such as heat stress and cold stress, several amino acids that are synthesized by animals can be considered as conditionally essential because their rates of catabolism are not compensated by rates of anabolism [14,15].

Based on evidence of the roles of proline in the maintenance of cell competence under environmental stress conditions, we investigated effects of dietary proline supplementation on haemato-biochemical attributes and semen quality indicators in rabbit bucks exposed to natural heat stress.

## 2. Experimental Section

### 2.1. Ethics Statement 

The present study was conducted in the Rabbit Research Unit, Department of Animal Production, Faculty of Agriculture, Zagazig University, Egypt. The procedures imposed on the animals were carried out meeting the International Guiding Principles for Biomedical Research Involving Animals [16] and according to the Directive 2010/63/EU of the European Parliament and of the Council of 22 September 2010 on the Protection of Animals Used for Scientific Purposes. In agreement with the Directive 2010/63/EU, the experimental procedures were previously assessed and approved by the INIA Committee of Ethics in Animal Research (report CEEA2014/087).

### 2.2. Animal Management and Experimental Groups

**Figure 1:**
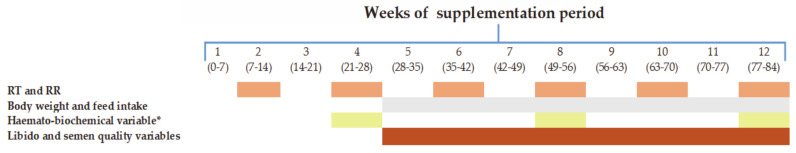
Diagram for the experimental design. RT= rectal temperature; RR= respiration rate; * Haematological variables were carried out at week 12 only.

Twenty-four New Zealand White 28-week-old adult rabbit bucks with an average body weight (BW) of 3.28 ± 0.18 kg were used for experiments. Bucks were kept individually in galvanized wire cage batteries of standard dimensions (40 × 50 × 35 cm) in a naturally ventilated rabbitry. Bucks were fed a basal pellet diet that covered all daily nutritional requirements (Table 1; NRC [17]) and were offered free access to clean tap water. Bucks were randomly categorized into four homogeneous experimental groups (*n* = 6) according to degrees of supplementation with dietary proline (L-Proline, chemical formula: C5H9NO2, CAS number: 147-85-3, molecular weight:115.13 g/mol, Sigma-Aldrich, Chemie GmbH, Eschenstrasse, 5 D-82024 TAUFKIRCHEN, Darmstadt, Germany). Bucks in the control group (P0) were fed the basal pellet diet with no supplements whereas, based on previous studies [12,13,14], bucks in the other three groups were fed basal pellet diets supplemented with proline at 50 (P50), 100 (P100), and 150 (P150) mg/kg dry matter (DM). Feeding treatments were given for 12 consecutive weeks (from July to September), and the first 4 weeks were considered as an adaptive period for the treatment (Figure 1).

### 2.3. Meteorological Variables

Ambient temperature and relative humidity were recorded daily during the entire experimental period using a hygrothermograph (ST-50A, SEKONIC, Tokyo, Japan) located in the animal facility. Records of ambient temperature and relative humidity of each day were used to estimate daily means for each variable. The THI was calculated using the following equation:

THI= db − [(0.31 − 0.31(RH)] × [(dbºC − 14.4)],

where db is the dry bulb temperature in Celsius and RH is the percentage relative humidity. The calculated THI values were subsequently classified as follows: <27.8 = absence of heat stress; 27.8 to 28.9 = moderate heat stress; 28.9 to 30.0 = severe heat stress, and >30.0 = extremely severe heat stress [19]. Records of daily photoperiods (daylight durations) were obtained from a nearby meteorological station.

### 2.4. Analyses of BW, Feed Intake, Rectal Temperature, and Respiratory Rate

BW and feed intake were recorded weekly for each buck (8 observations × 6 bucks; 48 observations per treatment). Rectal temperature and respiration rate were monitored and recorded every two weeks for each buck (weeks 2, 4, 6, 8, 10, and 12 of the experimental period; 6 collections × 6 bucks; 36 observations per treatment). 

### 2.5. Analyses of Haemobiochaemistry, Redox Status and Hormones

Blood samples were collected in heparinized tubes from the ear vein of each buck at weeks 4, 8, and 12 of the experimental period (3 collections × 6 bucks; 18 observations per treatment). A fraction of the blood samples collected at week 12 (6 observations/treatment) was used at once for counting red and white blood cells and platelets [20]. Haemoglobin contents were assessed colorimetrically using commercial kits (Biodiagnostic, Giza, Egypt). Immediately, plasma samples were obtained by centrifugation at 700× *g* for 20 min and were then stored at −20 °C until analyzed. Plasma samples were analyzed for total protein and albumin concentrations. Globulin concentrations were calculated by subtracting albumin values from the total protein values. Concentrations of creatinine, urea, triglycerides, total cholesterol, high-density lipoproteins (HDL), glucose, aspartate amino transferase (AST), and alanine amino transferase (ALT) were also determined. Blood plasma redox status indicators (in terms of total antioxidant capacity, TAC, and activity of malondialdehyde, MDA, and superoxide dismutase, SOD) were determined. All blood plasma biochemical variables and blood plasma redox status indicators were assayed using spectrophotometric procedures (Hitachi spectrophotometer, Japan) and commercial kits obtained from Biodiagnostic (Giza, Egypt). Concentrations of cortisol and testosterone in blood plasma were measured using enzyme immunoassay analysis. The lower limit of assay detection was 0.057 ng/mL for testosterone and 0.111 ng/mL for cortisol. The intra- and inter-assay CV were 6.73 and 8.90% for testosterone (Monobind Inc., Lake Forest, CA, USA) and 6.96 and 13.63% for cortisol (R&D System, Minneapolis University, Minneapolis, MN, USA).

### 2.6. Evaluation of Libido, Semen Quality, and Seminal Plasma Redox Status

Semen samples were collected weekly over 8 weeks (from weeks 4 to 12), so 48 evaluations of libido and semen quality variables were performed per treatment (8 ejaculates × 6 bucks). Reaction time (libido indicator) was determined by recording with a stopwatch the time from introducing a doe into the cage of a buck to achievement of complete ejaculation. Semen samples were collected using an artificial vagina and a teaser doe. Ejaculate volumes were recorded using a graduated collection tube after the removal of gel masses. Sperm concentration, progressive motility, viability, and morphology were evaluated following the guidelines of the International Rabbit Reproduction Group [21]. Sperm cell concentrations were determined after semen dilution (1:100) in formaldehyde phosphate buffered saline solution using an improved Neubauer chamber slide (GmbH + Co., Brandstwiete 4, 2000 Hamburg 11, Germany) and a light microscope (Olympus CH2CH-2; Olympus Optical Co. Ltd., Tokyo, Japan) at 40× magnification. Percentages of motile sperm (forward motility) were determined by visual examination in several microscopic fields for each semen ejaculate sample using a light microscope with a heated stage at 100× magnification with classification of subjective assessments ranging from 0% to 100%. Assessments of sperm cell viability and morphological abnormality were performed by counting 200 sperm cells stained with eosin–nigrosine blue staining mixture. Sperm cells with complete or partial staining were considered dead, whereas unstained sperm cells were considered viable. 

The ejaculate volume, sperm concentration, and percentages of motile, viable and normal morphology sperm cells were used to estimate total sperm output (TSO) = semen ejaculate volume (mL) × semen concentration (×10^6^/mL), total motile sperm (TMS) = percentage of motile sperm × total sperm output (×10^6^/ejaculate) and total functional sperm fraction (TFSF) = total sperm output (×10^6^/ejaculate) × percentage of progressive motility × percentage of normal sperm morphology. Percentages of sperm cells with intact acrosomes were evaluated in dried semen smears containing a total of 200 sperm cells stained with erythrosin B and naphthol yellow S using a light microscope (100× magnification). The sperm cells were classified according to the presence or absence of acrosome cap regions in their head regions (i.e., intact or non-intact acrosomes, respectively [22]).

Seminal plasma was obtained by centrifugation of semen samples at 700 ×g for 20 min. Samples were then stored at −20 °C until analysis. TAC and MDA concentrations were colorimetrically determined as indicators of seminal plasma redox status using commercially available kits (Biodiagnostic, Giza, Egypt).

### 2.7. Statistical Analysis

A MIXED procedure for repeated measurements (SAS, 2001, version 8, Cary, NC, USA [23]) was used for assessing body weight, feed intake, rectal temperature, respiratory rate, haemato-biochemical attributes, redox status indicators, hormones (cortisol and testosterone) semen physical characteristics and seminal plasma redox status as dependent variables. Levels of dietary proline (LDP) (0, 50, 100, and 150 mg/kg DM), time of sampling and/or data collection (from week 4 to 12 for libido and semen quality variables and weeks 4, 8, and 12 for biochemical variables) and their interactions were introduced as fixed independent variables, and individual buck identities were introduced as a random factor. Meteorological variables and haematological variables were analyzed using a general linear model procedure (one way analysis of variance; SAS, 2001). Differences among treatment groups were identified using Duncan’s new multiple range post-hoc test and were considered significant when *p* < 0.05. All results were expressed as least square means ± pooled standard error of mean (SEM). For determining a possible dose–response curve, the response of each dependent variable for different LDP (0, 50, 100, 150 mg/kg DM diet) were tested using orthogonal contrast statements for linear, quadratic, and cubic responses. Dose–response curves for significant relationships were created using a suitable regression equation to identify the optimum doses of dietary proline. Confidence intervals were estimated for each dose–response curve and the lowest doses of proline that resulted in the greatest responses (improvements or reductions) within the confidence intervals [23] and physiological range [24,25] of the dependent variable were considered the optimal doses.

## 3. Results

### 3.1. Meteorological Variables

Mean values of ambient temperature, relative humidity, THI, and daylight lengths (photoperiod) during the experimental period were 33.47 ± 0.40 °C, 75.17 ± 2.25%, 31.99 ± 0.37, and 12.80 ± 0.73 h, respectively (Table 2).

### 3.2. Analysis of BW, Feed Intake, Rectal Temperature, Respiration Rate, and Haematological Variables

**Figure 2:**
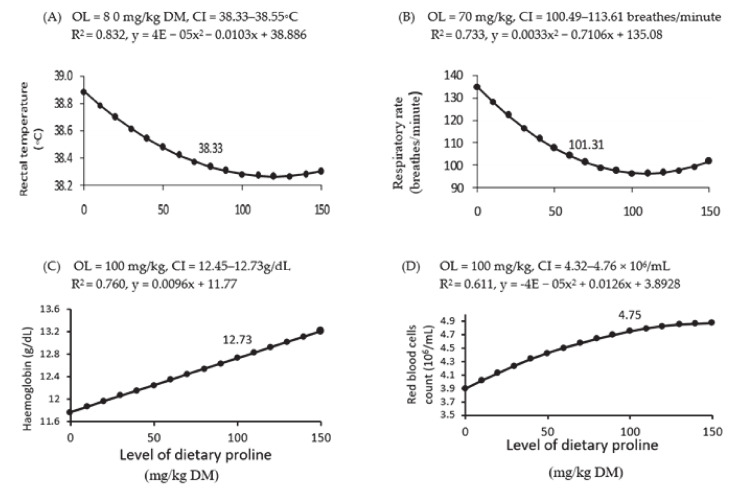
Dose–response curve of rectal temperature (**A**), respiratory rate (**B**), haemoglobin concentration (**C**), and red blood cell counts (**D**) for different levels of dietary supplemental proline. DM = dry matter, OL = optimum level of dietary supplemental proline and CI = confidence interval. R^2^ = regression value, and x and y are the dependent variable (proline level) and the independent variable of the regression equation, respectively. The figure only shows significant (*p* < 0.05) relationships.

Treatment with LDP did not affect BW and feed intake (Table 3). On the other hand, increases in LDP affected quadratically the rectal temperature (*p* = 0.009), respiratory rate (*p* < 0.001), and red blood cell count (*p* = 0.017) and linearly the haemoglobin concentration (*p* = 0.002) (Table 3). Dose–response curves showed that the optimal LDP for rectal temperatures and respiratory rates were established at 70 (Figure 2 A) and 80 (Figure 2 B) mg/kg DM, respectively, and for haemoglobin concentrations (Figure 2 C) and red blood cell counts (Figure 2 D) were found at 100 mg/kg DM. Conversely, increasing LDP did not affect mean corpuscular haemoglobin concentrations, white blood cell counts or platelet counts (*p* > 0.05; Table 3).

### 3.3. Analysis of Blood Plasma Biochaemicals, Redox Status, and Hormones

**Figure 3:**
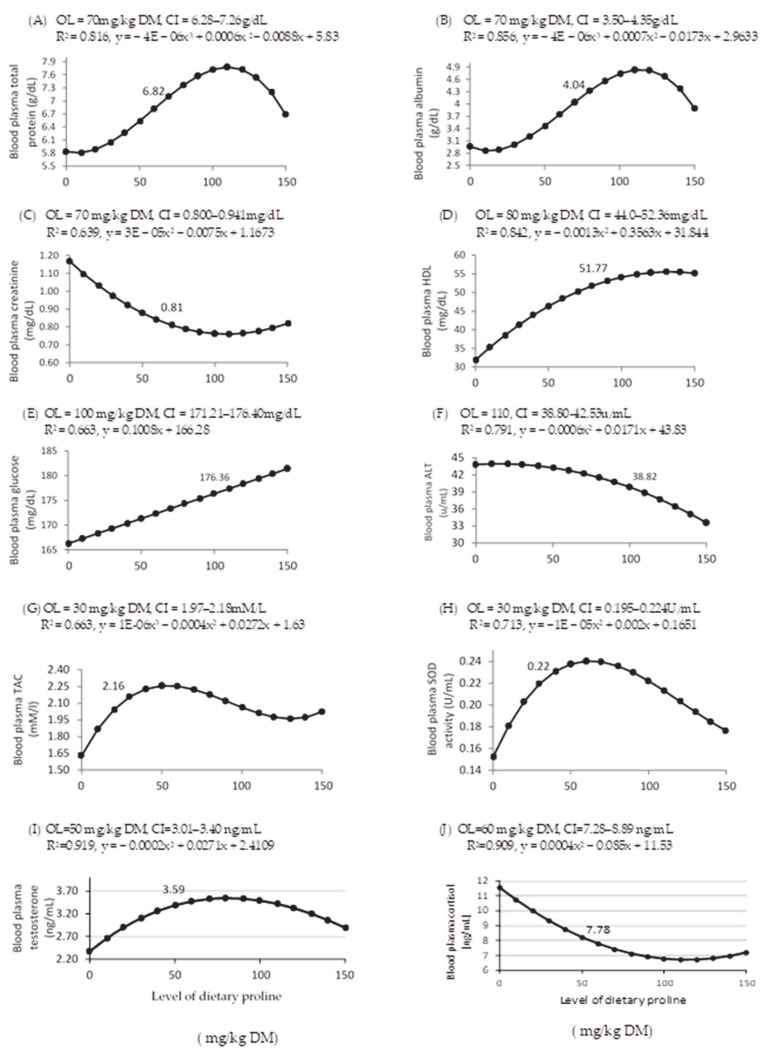
Dose–response curve of blood plasma total protein (**A**), albumin (**B**), creatinine (**C**), high density lipoprotein (HDL, (**D**)), glucose (**E**), alanine aminotransferase (ALT, (**F**)), total antioxidant capacity (TAC, (**G**)), superoxide dismutase activity (SOD, (**H**)), testosterone (**I**), and cortisol (**J**) for different levels of dietary supplemental proline. DM = dry matter, OL = optimum level of dietary supplemental proline and CI = confidence interval, R^2^ = regression value, and x and y are the dependent variable (proline level) and the independent variable of the regression equation, respectively. The figure only shows significant relationships (*p* < 0.05).

The effects of various LDP on blood plasma biochemical variables, redox status indicators, and hormones in rabbit bucks are shown in Table 4 and Figure 3. Increasing LDP cubically affected concentrations of blood plasma total protein (*p* = 0.006) and albumin (*p* = 0.008), and the optimal proline dose was 70 mg/kg DM (Figure 3A,B, respectively). Concentrations of blood plasma creatinine (*p* = 0.018; Table 4) and ALT (*p* = 0.005; Table 4) were quadratically related to increasing LDP, with optimal effects at 70 (Figure 3C) and 110 (Figure 3F) mg/kg DM, respectively (Figure 3). A quadratic relationship (*p* < 0.001) between dietary proline and blood plasma HDL concentrations was observed, with an optimal response at 80 mg/kg DM (Figure 3D), whereas the relationship between dietary proline and blood plasma glucose concentrations (*p* = 0.005) was linear and was optimal at 100 mg/kg DM (Figure 3E). Increasing LDP did not affect blood plasma concentrations of globulin, urea, triglycerides, total cholesterol, and AST. However, increasing LDP cubically affected blood plasma TAC (*p* = 0.041, Figure 3G) and SOD activity (*p* = 0.012, Figure 3H), with optimal responses to dietary proline at 30 mg/kg DM.

### 3.4. Evaluation of Libido, Semen Quality, and Seminal Plasma Redox Status

**Figure 4:**
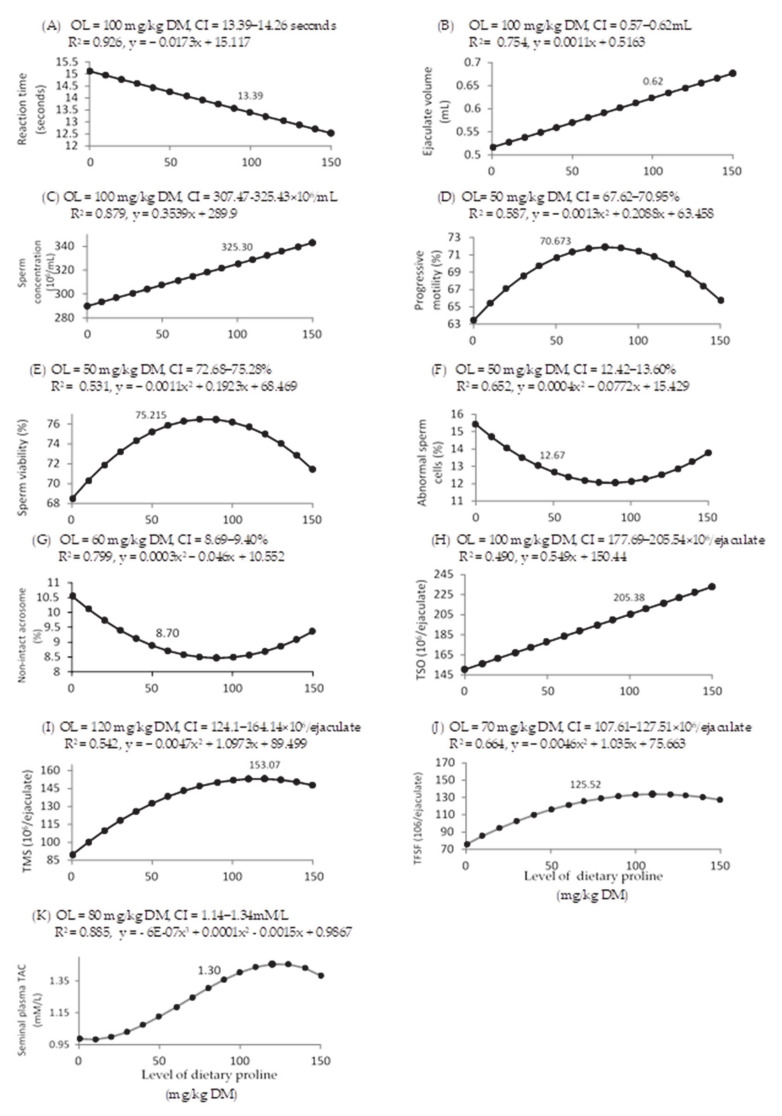
Dose–response curve of reaction time (**A**), ejaculate volumen (**B**), sperm concentration (**C**) and percentages of motile sperm cells (**D**), viable sperm cells (**E**), normal sperm cells (**F**), non-intact acrosome sperm cells (**G**), total sperm output (TSO, (**H**)), total motile sperm count (TMSO, (**I**)), total functional sperm fraction (TFSF, (**J**)), and seminal plasma antioxidant capacity (TAC, (**K**)) for different levels of dietary supplemental proline. DM = dry matter, OL = optimum level of dietary supplemental proline, CI = confidence interval, R^2^ = regression value, and x and y are the dependent variable (proline level) and the independent variable of the regression equation, respectively. The figure only shows significant (*p* < 0.05) relationships.

Increasing LDP also caused linear decreases in reaction times (*p* < 0.001) and linear increases in ejaculate volume, sperm concentration, and total sperm output (*p* < 0.001 for all; Table 5). Corresponding dose–response curves showed that the optimal LDP was at 100 mg/kg DM (Figure 4A–C). Percentages of motile, viable, abnormal, and non-intact acrosome sperm cells were quadratically affected by LDP. Optimal percentages of motile, viable and abnormal sperm cells were achieved with 50 mg/kg DM of proline (Figure 4D–F), whereas non-intact acrosome sperm cells were optimally improved at 60 mg/kg DM (Figure 4G). TSO was linearly affected by the LDP (*p* < 0.001; Table 5), with optimal response found at 100 mg/kg DM (Figure 4H). TMS and TFSF were quadratically affected by the LDP (*p* < 0.001; Table 5), with optimal proline concentrations of 120 and 70 mg/kg DM, respectively (Figure 4I,J).

The LDP supplementation quadratically affected blood plasma testosterone and cortisol concentrations (*p* < 0.001 for both, Table 4, with optimal responses at 50 and 60 mg/kg DM, respectively, Figure 3I,J, respectively) and cubically affected seminal plasma TAC (*p* = 0.006; Table 5), with an optimal response at 80 mg/kg DM (Figure 4K).

## 4. Discussion

There is increasing evidence of the role of proline as a biologically versatile functional amino acid, particularly of its functions under environmental stress conditions. Thus, in the present study, we assessed the potential of proline to enhance heat stress tolerance in rabbit bucks exposed to an average THI of around 31.99 (i.e., extremely severe heat stress conditions; [19,26]). We acknowledge the lack of a control group without exposition to heat stress but the negative effects of heat stress on physiology and metabolism of rabbits are already well-known and described and, in light of animal welfare, the inclusion of such a control group would have increased the number of rabbits in the experimental procedure. Dietary supplemental proline at a level range between 50 and 100 mg/kg DM reduced heat stress-related indicators like rectal temperatures, respiratory rates, and blood plasma cortisol concentrations [27], which suggests improvement in the heat tolerance of animals. Rabbits are physiologically adapted to lose body overheat by increasing panting rate (respiratory rate) as they have a low number of functional sweat glands [28]. In our study, the reduction in rectal temperature, however, was not accompanied by an increase in respiratory rate, indicating implications of other adaptive mechanisms.

The results also showed that increasing LDP up to 70 mg/kg DM improved blood plasma total protein and albumin concentrations. This could be mediated by the proteinogenic and chaperone properties of proline [4]. In addition, albumin and proline have demonstrated osmoprotective properties in plasma and cells [1,3]. This property is crucial under environmental heat stress conditions since it regulates water balance and facilitates the maintenance of protein and enzyme stability. It is worth noting that the relationship between proline levels and either total blood plasma protein levels or blood plasma albumin levels was cubic. In fact, these relationships are difficult to interpret, however they at least highlight the biological complexity between these variables and the importance of adjusting proline level supplementation to achieve desired biological effects. On the other hand, increasing LDP up to 110 mg/kg DM decreased blood plasma ALT activity. High extracellular ALT activity is considered a negative indicator of liver function [1], thus proline might have a hepatic-protective effect. Also, increasing LDP up to 100 mg/kg DM improved red blood cell counts and haemoglobin concentrations. These enhancements are also required adaptive mechanisms during heat stress to improve oxygen circulation during panting process, improving the panting process efficiency and thus heat loss [27].

Heat stress impairs energy status and energy utilization in rabbits [29]. It was reported that availability of blood plasma glucose during heat stress allows energy utilization with lower metabolic heat production compared to other energy-yielding nutrients [1,27]. Glucose is the main source of electrons for ATP and NADPH production in most mammalian cells, and it is a glycerol precursor and intermediate for nonessential amino acid synthesis [29]. Interestingly, increasing LDP up to 100 mg/kg DM increased blood plasma glucose concentrations, which may be ascribed to the contribution of proline to gluconeogenesis [30], the regulation of proline-dependent cellular energy signaling pathways, such as the rapamycin activation pathway [8], which integrates signals from nutrients such as glucose and amino acids [9], and/or improved cellular glucose uptake due to proline serving as a structural component [31,32] or inducer of genes encoding glucose transporters (GLUT; [33]).

Proline supplementation improved the redox status of naturally heat-stressed rabbit bucks by increasing TAC and SOD activity in blood plasma. However, these effects on redox status were only observed with low proline concentrations (30 mg/kg DM), and the relationship was cubic. Proline also has oxygen radical scavenging activities that may contribute to these observations [7,13]. However, conversion of proline into pyrroline-5-carboxylate (p5C) by the PRODH/POX catalyzing system in the inner mitochondrial membrane generates superoxide anions, which participate in ROS-dependent apoptotic pathways [6]. These diverse effects of proline suggest that a complex relationship between proline supplementation and redox status and further specific studies are therefore necessary. According to our results, low proline concentrations could improve the redox status by improving TAC and SOD activity, whereas high proline concentrations may act as a source of free radicals.

Finally, assessment of libido and spermatogenesis after dietary proline supplementation showed linear improvements in libido, semen ejaculate volume, and sperm concentration. These results highlight the positive effects of proline on reproductive traits. In bucks, the major facilitative neurotransmitters that control sexual motivation and copulatory performance are dopamine, glutamate, and norepinephrine [34]. In addition, a high-affinity L-proline transporter is expressed in mammalian brain tissues, where it mediates high-affinity uptake of neurotransmitters, including norepinephrine, dopamine, serotonin, glutamate, and glycine [35]. Proline is also a dietary precursor for the amino acid arginine [36], which contributes to nitric oxide (NO) synthesis. NO has been previously shown to improve libido and erectile function by increasing blood flow throughout the genital tract [37]. Thus, the present effects of dietary proline supplementation may be related to sexual neurotransmitter signaling pathways and blood flow throughout the genital tract, leading to improvements in the overall sexual performance of rabbit bucks. Besides, proline contributes to multiple signaling pathways that are involved in the molecular regulation of spermatogenesis process. For example, in Sertoli cells, the activation of proline rich regions of androgen receptors (amino acids 352–359) associating with the SH3 domain of Src has been found to be a critical pathway for maintaining spermatogenesis [38]. Also, proline has been found to be a specific component of seminal plasma and germ cell proteins. For example, proline is a major structural amino acid component of nucleoproteins and polyamines [13], which are required extensively during mitosis and meiosis in male germ cells [37]. Among such proteins, FNDC3A is a proline-rich amino-terminal protein that, as an integral membrane protein in male germ cells, may contribute to spermatogenesis by mediating the mandatory adhesion between Sertoli cells and round spermatids [39]. 

The enhancements in libido and spermatogenesis found in proline-supplemented rabbit bucks may be also attributed to improved blood plasma testosterone concentrations (steroidogenesis). Testosterone is required for several critical processes during spermatogenesis including maintenance of the blood-testis barrier, meiosis, Sertoli-spermatid adhesion and sperm release as well as sexual activity [38,40]. Again, proline is a structural component of a specific family of proline-directed serine-threonine kinases known as mitogen-activated protein kinases (MAPKs) including three major subfamilies: p38, ERK, and JNK. These kinases are important cellular signaling components that are involved in the regulation of steroidogenesis in germ cells of mammals [41].

In this study, dietary proline supplementation improved sperm motility, viability, morphology, and acrosome integrity. These enhancements were dose-dependent (quadratic relationship), although high doses of more than 50–60 mg/kg DM caused adverse effects. Collectively, these results highlight the concentration-dependent dual roles of proline. In support of this notion, cycling between proline and its product P5C provides a redox shuttle, and the electrons from proline can be used to generate ATP or free radicals. Thus, the energy obtained from proline degradation may maintain survival or stimulate a poptosis [6,42]. In this context, Yen et al. [43] reported that the continuous accumulation of P5C, which is highly unstable and toxic, leads to redox imbalance and impairs sperm cell function. Given the present heat stress conditions, which typically induce the activation of oxidative stress pathways, the effects of the free radicals generated by proline catabolism may be amplified, which makes sperm cells more sensitive to proline concentration. Generally, these results are inconsistent with those obtained in previous studies, in which high proline intake inhibited enzymes such as acetyl cholin esterase, Na+-, K+-ATPase, and aminotransferase [43,44] and promoted energy deficit [45] and oxidative stress [43].

## 5. Conclusions

The present study revealed the usefulness of dietary proline supplementation for enhancing heat-tolerance and semen quality of rabbit bucks affected by heat-stress. These effects are dose-dependent and were achieved at doses ranging between 50 and 100 mg proline/kg DM. Higher proline concentrations likely have undesirable effects on redox status and sperm quality. These results pave the way for future studies on effects and mechanisms of proline supplementation on animal performance under different stress circumstances. 

## Figures and Tables

**Table 1:** Proximate analysis of basal diet components expressed as percentages of dry matter (DM).

Items	Basal Diet
Ingredients(g/kg DM)	
Berseem clover hay	30
Soybean meal	20
Maize	20
Wheat bran	16
Barley grain	10
Molasses	2
Limestone	1
NaCl	0.5
Premix ^†^	0.5
Analyzed composition (%, on DM basis) ^‡^	
Crude protein	16.54
Ether extract	2.25
Crude fiber	12.33
Dry matter	88.06
Organic matter	90.57
Ash	9.43
Nitrogen-free extract	59.45
Calculated composition%	
ME, MJ/kg ^§^	7.95
Calcium	0.88
Available phosphorus	0.20

^†^ Each 1 kg of premix (minerals and vitamins mixture) contains vit. A, 20,000 IU; vit. D3, 15,000 IU; vit. E, 8.33 g; vit. K, 0.33 g; vit. B1, 0.33; vit. B2, 1.0 g; vit. B6, 0.33 g; vit. B5, 8.33 g; vit. B12, 1.7 mg; pantothenic acid, 3.33 g; biotin, 33 mg; folic acid, 0.83 g; and choline chloride, 200 g. ^‡^ Chemical analysis according to AOAC [18]. ^§^ Calculated according to NRC [17].

**Table 2:** Experimental ambient temperature, relative humidity, temperature–humidity index (THI), and photoperiod; data are presented as means ± standard errors of the mean (SEM).

Parameter	July	August	September	Overall	SEM	*p*-Value
Ambient temperature (°C)	34.63 ^a^	33.43 ^ab^	32.35 ^b^	33.47	0.40	0.007
Relative humidity (%)	74.50	72.50	78.50	75.17	2.25	0.125
THI	33.03 ^a^	31.80 ^b^	31.16 ^b^	31.99	0.37	0.014
Photoperiod (h)	13.24 ^a^	12.76 ^b^	12.41 ^b^	12.80	0.73	0.016

Mean values followed by different superscript letters in the same row are significantly different (*p* < 0.05).

**Table 3:** Concentration-dependent effects of proline supplements on body weight, feed intake, rectal temperature, respiration rate, and haematological attributes of rabbit bucks during the experimental period; data are expressed as least square means ± standard errors of the mean (SEM).

Variable ^†^	Proline Concentration ^‡^				SEM	*p*-Value ^§^			
						T	Contrast		
	P0	P50	P100	P150			L	Q	C
Initial body weight (kg)	3.39	3.44	3.41	3.40	0.012	0.951	-	-	-
Body weight (kg)	3.61	3.66	3.60	3.61	0.080	0.950	0.618	0.763	0.892
Feed intake (g/day)	178.54	185.50	176.67	181.25	2.26	0.200	0.618	0.763	0.892
RT (◦C)	38.91 ^a^	38.43 ^b^	38.38 ^b^	38.70 ^ab^	0.034	<0.001	0.527	0.009	0.102
RR (breaths/minute)	135.67 ^a^	105.94 ^b^	98.39 ^c^	101.28 ^bc^	2.38	<0.001	0.081	<0.001	0.277
Haematological attributes									
RBC (10^6^/mL)	3.89 ^b^	4.44 ^ab^	4.73 ^a^	4.88 ^a^	0.173	0.047	0.102	0.017	0.917
Hb (g/dL)	10.69 ^b^	13.75 ^a^	12.98 ^a^	12.55 ^a^	0.445	0.007	0.002	0.259	0.191
MCH (pg)	27.51	31.14	27.44	25.98	0.112	0.178	0.201	0.122	0.271
WBC (10^3^/mL)	6.77	3.76	4.60	4.73	0.636	0.178	0.094	0.518	0.413
Platelets	711.01	889.33	925.04	878.33	23.16	0.132	0.217	0.130	0.271

Mean values followed by different superscript letters in the same row are significantly different (*p* < 0.05). ^†^ RT, rectal temperature; RR, respiration rate; RBC, red blood cells; Hb, haemoglobin; MCH, mean corpuscular haemoglobin (Hb × 10/RBC); and WBC, white blood cells. ^‡^ P0, P50, P100, and P150 indicate 0-, 50-, 100-, and 150-mg proline/kg DM; respectively. ^§^ T, treatment; L, linear response; Q, quadratic response; C, cubic response.

**Table 4:** Concentration-dependent effects of proline on biochemical variables, redox status indicators, and hormones in rabbit bucks; data are expressed as least square means ± standard errors of the mean (SEM).

Variable ^†^	Proline Concentration ^‡^				SEM	*p*-Value ^§^			
						T	Contrast		
	P0	P50	P100	P150			L	Q	C
Biochemical variables									
TP (g/dl)	5.83 ^c^	6.537 ^bc^	7.72 ^a^	6.70 ^b^	0.16	0.003	0.315	0.412	0.006
Albumin (g/dL)	2.96 ^c^	3.47 ^bc^	4.14 ^a^	3.91 ^b^	0.064	<0.001	0.464	0.241	0.008
Globulin (g/dL)	2.87	3.07	2.98	2.80	0.20	0.607	0.539	0.606	0.298
Creatinine (mg/dL)	1.16 ^ab^	0.90 ^c^	0.74 ^c^	0.83 ^bc^	0.08	0.035	0.199	0.018	0.200
Urea(mg/dL)	27.00	23.38	22.14	21.92	2.43	0.237	0.224	0.132	0.734
TG (mg/dL)	94.00	87.21	88.67	99.88	1.79	0.296	0.678	0.463	0.094
TC (mg/dL)	74.11	73.84	78.43	80.88	5.66	0.621	0.935	0.228	0.706
HDL(mg/dL)	34.64 ^b^	37.93 ^b^	62.47 ^a^	52.38 ^a^	2.39	0.001	0.990	<0.001	0.261
Glucose (mg/dL)	143.56 ^c^	212.31 ^ab^	162.52 ^c^	176.96 ^bc^	5.34	0.027	0.005	0.536	0.665
AST (U/mL)	44.02	42.70	40.41	33.40	3.47	0.248	0.521	0.115	0.262
ALT (U/mL)	79.88 ^a^	60.87 ^b^	57.15 ^b^	56.59 ^b^	1.96	0.004	0.241	0.005	0.295
Redox status indicators Redox status indicators									
TAC (mM/L)	1.63 ^b^	2.26 ^a^	2.06 ^a^	2.03 ^ab^	0.095	0.002	0.267	0.195	0.041
MDA (nmol/mL)	13.30	12.12	11.71	11.83	0.277	0.051	0.168	0.132	0.373
SOD (U/mL)	0.152 ^b^	0.237 ^a^	0.222 ^a^	0.176 ^a^	0.012	0.015	0.160	0.281	0.012
Hormones									
Testosterone (ng/mL)	2.37 ^c^	3.39 ^a^	3.49 ^a^	2.59 ^b^	0.035	<0.001	0.247	<0.001	0.140
Cortisol (ng/mL)	11.58 ^a^	8.01 ^b^	6.92 ^b^	8.15 ^ab^	0.138	<0.001	0.312	<0.001	0.094

Mean values followed by different superscript letters in the same row are significantly different (*p* < 0.05). ^†^ TP, total protein; TG, Triglycerides; TC, total cholesterol; AST, aspartate transaminase; ALT, alanine transaminase; TAC, total antioxidant capacity; MDA, malondialdehyde; SOD, superoxide dismutase. ^‡^ P0, P50, P100, and P150 indicate 0-, 50-, 100-, and 150-mg proline/kg DM; respectively. ^§^ T, treatment; L, linear response; Q, quadratic response; C, cubic response.

**Table 5:** Concentration-dependent effects of proline supplements on reaction times (libido), semen physical variables, and seminal plasma redox status indicators in rabbit bucks; data are expressed as least square means ± standard errors of the mean (SEM).

Variable ^†^	Proline Concentration ^‡^				SEM	*p*-Value ^§^			
						T	Contrast		
	P0	P50	P100	P150			L	Q	C
Reaction time(s)	15.46 ^a^	13.71 ^b^	12.46 ^b^	12.67 ^b^	0.371	<0.001	<0.001	0.213	0.236
Physical variables									
Volume (mL)	0.51 ^b^	0.58 ^b^	0.65 ^a^	0.66 ^a^	0.031	0.002	<0.001	0.314	0.607
SCon (10^6^/mL)	278.96 ^b^	323.33 ^a^	326.67 ^a^	336.83 ^a^	8.121	<0.001	<0.001	0.239	0.193
Progressive motility (%)	63.33 ^b^	69.04 ^a^	71.04 ^a^	65.83 ^ab^	1.297	<0.001	0.201	<0.001	0.668
Live sperm (%)	68.33 ^b^	75.63 ^a^	76.83 ^a^	71.67 ^ab^	1.552	0.003	0.147	<0.001	0.697
Abnormal sperm (%)	15.54 ^a^	12.33 ^b^	11.46 ^b^	13.67 ^ab^	0.514	<0.001	0.200	<0.001	0.332
Non-intact acrosome (%)	10.63 ^a^	8.97 ^b^	8.71 ^b^	9.29 ^ab^	0.380	0.002	0.230	0.002	0.394
TSO (10^6^/ejaculate)	141.27 ^ab^	187.35 ^a^	213.89 ^a^	223.93 ^a^	14.63	<0.001	<0.001	0.223	0.963
TMS (10^6^/ejaculate)	89.49 ^b^	132.54 ^a^	151.93 ^a^	147.66 ^a^	9.60	<0.001	0.273	0.017	0.999
TFSF (10^6^/ejaculate)	75.61 ^b^	116.06 ^a^	132.92 ^a^	127.29 ^a^	8.37	<0.001	0.097	0.008	0.977
Redox status indicators									
TAC (mM/l)	0.987 ^c^	1.127 ^b^	1.40 ^a^	1.38^ab^	0.023	<0.001	0.240	0.340	0.006
MDA(nmol/mL)	14.91	12.83	13.27	13.17	0.398	0.096	0.061	0.847	0.727

Mean values followed by different superscript letters in the same row are significantly different (*p* < 0.05). ^†^ SCon, sperm concentration; TSO, total sperm output = semen ejaculate volume (mL) × semen concentration (10^6^/mL); TMS, total motile sperm = percentage of motile sperm × total sperm output (10^6^/ejaculate); TFSF, total functional sperm fraction = total sperm output (10^6^/ejaculate) × percentage of forward motility × percentage of normal sperm morphology TAC, total antioxidant capacity; MDA, malondialdehyde. ^‡^ P0, P50, P100, and P150 indicate 0-, 50-, 100-, and 150-mg proline/kg DM; respectively. ^§^ T, treatment; L, linear response; Q, quadratic response; C, cubic response.

## Data Availability

The data presented in this study are available on request from the corresponding author. The data are not publicly available due to privacy.
